# Employing Spectral Analysis to Obtain Dispersion Parameters in an Atmospheric Environment Driven by a Mesoscale Downslope Windstorm

**DOI:** 10.3390/ijerph182413027

**Published:** 2021-12-10

**Authors:** Cinara Ewerling da Rosa, Michel Stefanello, Silvana Maldaner, Douglas Stefanello Facco, Débora Regina Roberti, Tiziano Tirabassi, Gervásio Annes Degrazia

**Affiliations:** 1 Departamento de Física, Universidade Federal de Santa Maria, Santa Maria 97105-900, RS, Brazil; michelstefanello@gmail.com (M.S.); silvana.maldaner@gmail.com (S.M.); debora@ufsm.br (D.R.R.); tiziano.tirabassi@gmail.com (T.T.); gervasiodegrazia@gmail.com (G.A.D.); 2 Instituto Federal Farroupilha, São Vicente do Sul 97420-000, RS, Brazil; 3 Research Center on Remote Sensing and Meteorology, Universidade Federal do Rio Grande do Sul, Porto Alegre 91501-970, RS, Brazil; douglas.s.facco@gmail.com

**Keywords:** Vento Norte, downslope windstorm, neutral boundary layer, shear-driven turbulence, turbulent parameterization

## Abstract

Considering the influence of the downslope windstorm called “Vento Norte” (VNOR; Portuguese for “North Wind”) in planetary boundary layer turbulent features, a new set of turbulent parameterizations, which are to be used in atmospheric dispersion models, has been derived. Taylor’s statistical diffusion theory, velocity spectra obtained at four levels (3, 6, 14, and 30 m) in a micrometeorological tower, and the energy-containing eddy scales are used to calculate neutral planetary boundary layer turbulent parameters. Vertical profile formulations of the wind velocity variances and Lagrangian decorrelation time scales are proposed, and to validate this new parameterization, it is applied in a Lagrangian Stochastic Particle Dispersion Model to simulate the Prairie Grass concentration experiments. The simulated concentration results were shown to agree with those observed.

## 1. Introduction

Contaminant transport and dispersion in the planetary boundary layer is controlled by the mean wind flow and turbulent velocity components, respectively. The mean wind flow is associated with the direction of the mean transport of the contaminants, while the turbulence intensity drives the spread of the contaminant plume [1,2,3]. Therefore, the distinct Eulerian and Lagrangian traditional dispersion models must incorporate the physical parameters that represent the mean transport and dispersion effects into their formulation. Usually, Eulerian models employ eddy diffusivities to parameterize the concentration of turbulent fluxes [4], while Lagrangian models generally use turbulent velocity variances and decorrelation local time scales [5]. For both Eulerian and Lagrangian dispersion models, the mean wind flow must be specified.

The wind field and turbulent parameterizations are obtained from different meteorological scales. Therefore, detailed meteorological observations are of relevant importance in contaminant dispersion modeling studies. In this case, in addition to having the wind field originated by a mean flow, it is necessary to measure the high-frequency motions associated with the turbulent fluctuations, which are ultimately responsible for the diffusion.

In the surface boundary layer, turbulence is mainly driven by thermal and shear force phenomena. At the same time, the local topography can also reinforce the mean wind speed and consequently the turbulent activity in the presence of a downstream process provoked by topographic features [6,7,8,9,10].

**Figure 1:**
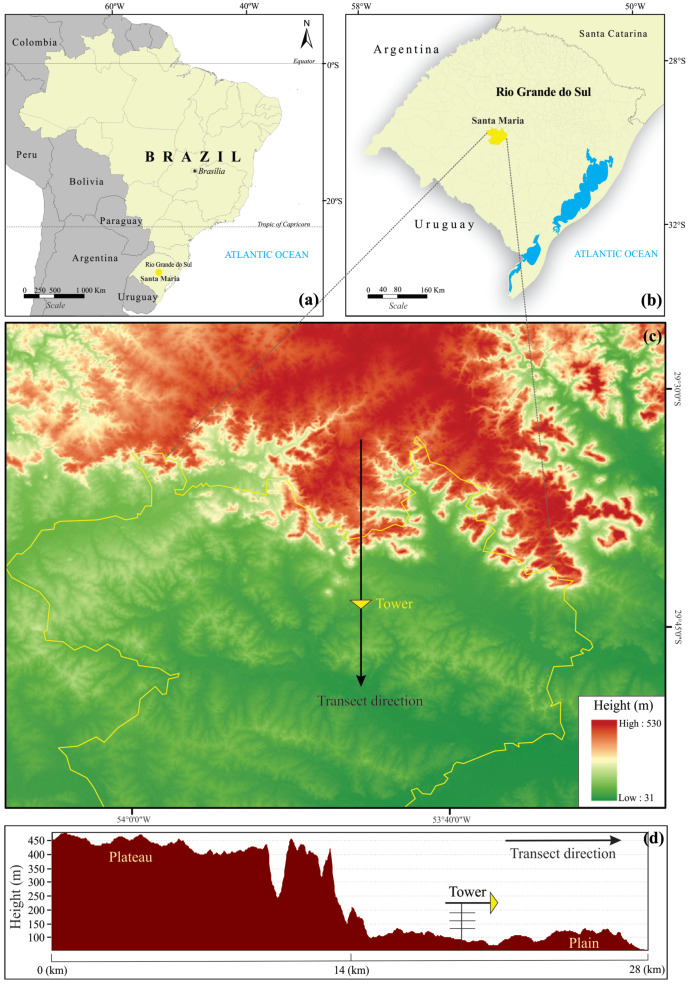
(**a**) Location of Santa Maria (yellow) in South America, (**b**) regional position of Santa Maria in Rio Grande do Sul State, (**c**) topography of the area surrounding the experimental site of Santa Maria, which is indicated by a yellow inverted triangle, and (**d**) topographical profile along the cross-section as indicated by the black line in (**c**). The arrow indicates the direction along the downslope.

In southern Brazil, there is a topographically forced airflow type that occurs in the cold season known as “Vento Norte” (VNOR; Portuguese for “North Wind” [11,12,13,14]). This mesoscale meteorological phenomenon generates an intense advection of warm air northward and produces a neutral boundary layer [13]. The VNOR windstorm is sustained by a specific large-scale atmospheric pressure system and induced by an abrupt difference in height (400 m), characterizing the plateau–plain interface in central Rio Grande do Sul State (Figure 1).

In this study, spectral Taylor’s statistical diffusion theory and turbulence data measured from a multiple-level micrometeorological tower were employed to derive the dispersion parameters under VNOR neutral conditions. Additionally, this turbulent parameterization is used in a Lagrangian stochastical dispersion model to simulate the ground-level contaminant concentration measured in the Prairie Grass dispersion experiment.

## 2. Experimental Site and Data Analysis

The field observations analyzed in this study refer to measurements collected from May to August of 2020 at the site of the Federal University of Santa Maria, southern Brazil (29${}^{\circ }$43${}^{\prime }$27.5${}^{\prime \prime }$ S, 53${}^{\circ }$45${}^{\prime }$36.1${}^{\prime \prime }$ W; elevation 88 m). The site is situated near Santa Maria (Figure 1a,b) in a 24 ha area covered by natural vegetation, which represents a typical environment of the Pampa Biome.

A modest zonally–oriented depression, which defines the transition of the plateau (to the north) from the prairie (to the south), is situated at a horizontal distance of ≈8 km just to the north of the experimental site. Such topography declivity is characterized by an elevation of 400 m (Figure 1c,d). More details about the site can be found in Rubert et al. (2018) [15].

Starting in December 2019, wind velocity components (*u*, *v*, *w*) and temperature (*T*) were measured at multiple levels in a 30 m micrometeorological tower. Turbulence measured at a frequency of 10 Hz was sampled by one IRGASON (Campbell Sci., Inc., Logan, UT, USA) 3 m, above the ground, and at 6 and 14 m by two CSAT3B (Campbell Sci., Inc., Logan, UT, USA) sonic anemometers, as well as by one CSAT3 (Campbell Sci., Inc., Logan, UT, USA) sonic anemometer at the height of 30 m. The wind data were first rotated to the preferential wind direction by applying a double rotation for each 1 h time series. Then, mean and turbulent quantities and energy spectra were obtained.

### Turbulent and Meteorological Variables of the VNOR

The dataset was initially verified to objectively individuate the VNOR episodes following, simultaneously, the subsequent criteria [13,16]: (i) wind speed above 3.0 m·s${}^{-1}$, (ii) wind direction ranging between 270${}^{\circ }$ (west–northwest) and 60${}^{\circ }$ (north–northeast), (iii) air temperature above the 90th percentile of the respective time and month, and (iv) minimum duration of 4 consecutive hours. Then, the time series of the above-mentioned variables was inspected by utilizing knowledge of the typical observed behavior of the atmospheric variables during an event [13,14,17]. The results presented hereinafter are related to 20 VNOR events and correspond to 116 h under such conditions obtained in the colder months of 2020 (May to August). The life cycle of the VNOR events varied from 4 to 17 h.

**Figure 2:**
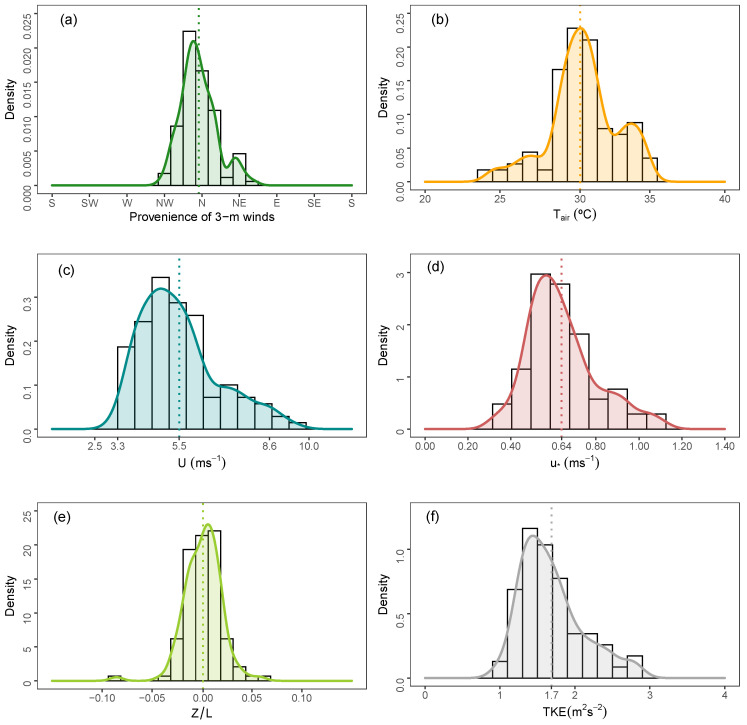
Density distributions at 3 m of (**a**) wind direction, (**b**) air temperature, (**c**) wind speed, (**d**) friction velocity, (**e**) stability parameter, and (**f**) TKE from May to August of 2020 relative to the VNOR cases. Dashed lines represent the means of the respective parameters.

Figure 2 presents the observed distributions at 3 m of the following micrometeorological variables: the wind direction, mean temperature ($T_{air}$), mean wind speed (*U*), stability parameter (z/L, being *z* the height (3 m) and *L* the Obukhov length), friction velocity ($u_{*}$), and turbulent kinetic energy (TKE). These distributions highlight the presence of the VNOR features being dominant wind direction from the north (Figure 2a), warm airflow (Figure 2b), and intense mean wind speed (Figure 2c). Figure 2d,e presents values of $u_{*}\approx 0.64$ and z/L→0 and characterizes a neutral shear-driven surface boundary layer.

The synoptic-scale system establishes the background environment necessary for VNOR development and evolution [13,14]. The combination of this background environment with terrain features is responsible for persistent and warm northerly winds of moderate-to-strong magnitudes that generate a neutral boundary layer, with z/L→0 for all measurement heights during the VNOR events. As shown in Figure 1, the terrain is reasonably uniform over a significant area around the measurements. Therefore, under this condition, it is expected that a neutral surface boundary layer be in equilibrium with the forcing responsible for its generation or intensification. The mean values of the meteorological variables along with the VNOR events are summarized in Table 1.

## 3. Turbulent Parameterization

In this section, mathematical formulations that allowed us to calculate the various dispersion parameters are presented. The following expression derived by Degrazia et al. (2000) [18] represents a model used to describe observed Eulerian velocity spectra ($S_{i}$) in a shear-driven neutral planetary boundary layer

(1) $$\frac{nS_{i}}{u_{*}^{2}}=\frac{1.5c_{i}\phi_{\epsilon }^{2/3}f}{(1+\frac{1.5f^{5/3}}{{\left[{\left(f_{m}\right)}_{i}\right]}^{5/3}}){\left[{\left(f_{m}\right)}_{i}\right]}^{5/3}}$$

where i=u,v,w, f=nz/U is the normalized frequency (*n* being the cyclic frequency in Hz and *z* the observation height), $\phi_{\epsilon }$$=\epsilon \kappa z/u_{*}^{3}$ is the dimensionless turbulent dissipation rate (ϵ is the mean turbulent kinetic energy dissipation per unit time per unit mass of fluid, and κ=0.4 is the von Kármán constant), ${\left(f_{m}\right)}_{i}$ is the non-dimensional frequency of the neutral spectral peak, $c_{i}=\alpha_{i}\alpha_{u}{\left(2\pi \kappa \right)}^{-2/3}$ with $\alpha_{u}=0.5\pm 0.05$, and $\alpha_{i}=1,4/3,4/3$ [19,20].

Using the residue theorem [21], Equation (1) can be integrated over the whole frequency domain to obtain the turbulent velocity variances

(2) $$\sigma_{i}^{2}=\frac{2.32c_{i}\phi_{\epsilon }^{2/3}u_{*}^{2}}{{\left(f_{m}\right)}_{i}^{2/3}}.$$

Applying the filtering procedure in the normalized Eulerian energy spectrum ($F_{i}^{E}$) to select the flow main energy-containing eddies results in [18]

(3) $$F_{i}^{E}\left(n\to 0\right)=\frac{S_{i}\left(n\to 0\right)}{\sigma_{i}^{2}}=\frac{0.64z}{{\left(f_{m}\right)}_{i}U}.$$

Based on the spectral Taylor’s statistical diffusion theory, the local decorrelation time scales ($T_{L_{i}}$) can be written as

(4) $$T_{L_{i}}=\frac{\beta_{i}F_{i}^{E}\left(n\to 0\right)}{4}$$

where $\beta_{i}=0.55U/\sigma_{i}$ is the ratio of the Lagrangian to the Eulerian decorrelation time scales [22,23,24].

Employing Equation (3) into Equation (4) yields

(5) $$T_{L_{i}}=0.088\frac{z}{\sigma_{i}{\left(f_{m}\right)}_{i}}.$$

Therefore, to estimate the time scales, it is necessary to provide the observed values of $\sigma_{i}$ and ${\left(f_{m}\right)}_{i}$.

## 4. Results and Discussion

**Figure 3:**
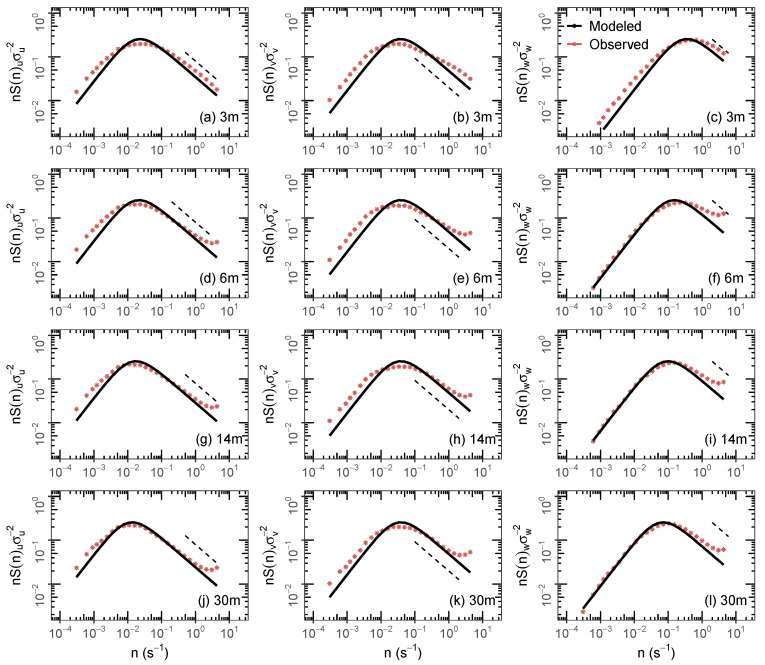
Representative spectra of the (**a**,**d**,**g**,**j**) longitudinal, (**b**,**e**,**h**,**k**) lateral, and (**c**,**f**,**i**,**l**) vertical turbulent velocity of the VNOR for heights 3 m (**a**–**c**), 6 m (**d**–**f**), 14 m (**g**–**i**,) and 30 m (**j**–**l**).

The turbulent velocity energy spectra describe how the velocity variances are distributed between the distinct frequencies [2]. In this study, the employed data set used to generate the energy spectral curves corresponds to 116 h of VNOR observations. Firstly, the linear detrending was applied in a 1 h time series to remove non-stationarity. Then, for such time window, the single spectra were calculated by employing the fast Fourier transform technique. The ensemble averages were computed to obtain multiple-level representative spectra; these averages of normalized spectra are shown in Figure 3. The observed wind velocity spectra (red asterisk) follow the Kolmogorov inertial subrange behavior [25] (black dashed line). The spectra for the *u* and *v* velocity components present an energetic peak in the turbulent region and decrease at low-frequency ranges for all height. Such a pattern observed in the horizontal velocity components is provoked by the high-mean wind velocity observed during VNOR hours (Table 1). The model, as given by Equation (1), effectively reproduces the observed spectra in the turbulent frequency range (continuous black line). Therefore, a good comparison between the observed and modeled spectral curves allows realistic decorrelation time scales and turbulent velocity variances to be obtained. To parameterize the dispersion processes in the numerical models, it is important to describe the scales of the energy-containing eddies. From the statistical point of view, these energetic eddies are responsible for the turbulent diffusive action, and its scales are characterized by the peak frequencies clearly identified in the spectral curves.

**Figure 4:**
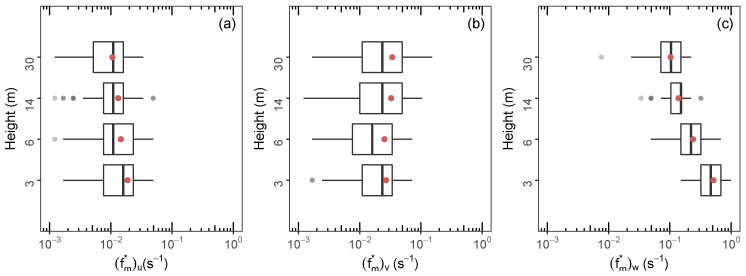
Boxplot of the dimensional frequency where the average spectra have their maximum ($f_{m}^{*}$) (**a**) longitudinal, (**b**) lateral and (**c**) vertical of the VNOR for heights 3, 6, 14 and 30 m. The means are shown as red dots. Outliers are shown by gray dots.

Figure 4 exhibits the vertical variation of the dimensional peak frequencies ($f_{m}^{*}$)${}_{i}$ for the wind velocity components. It can be seen that, for the *u* (Figure 4a) and *w* (Figure 4c) components, ${\left(f_{m}^{*}\right)}_{u}$ and ${\left(f_{m}^{*}\right)}_{w}$ decrease to lower frequencies as the height increases. This behavior is more evident in the vertical wind velocity component. As the height increases, the surface impedance effect decreases; as a consequence, the vertical eddy scales assume larger values. Different ${\left(f_{m}^{*}\right)}_{v}$ values tend to a nearly constant value (Figure 4b). This behavior is in qualitative agreement with ($f_{m}^{*}$)${}_{i}$ values obtained near the surface at a coastal site in southeastern Brazil by Martins et al. (2018) [26].

The dimensionless ratios of the velocity standard deviations ($\sigma_{i}$) to the friction velocity, which are employed to evaluate the surface boundary layer turbulence intensity, are presented in Table 2. The ratio values calculated from Equation (2) (M), employing the mean value of $\phi_{\epsilon }∼1.1$ and the distinct values of ${\left(f_{m}\right)}_{i}$ for each turbulent velocity component, are compared with the observed results (O). One can see that there is good agreement between the modeled and observed values as well as with previous studies by [2,17].

**Figure 5:**
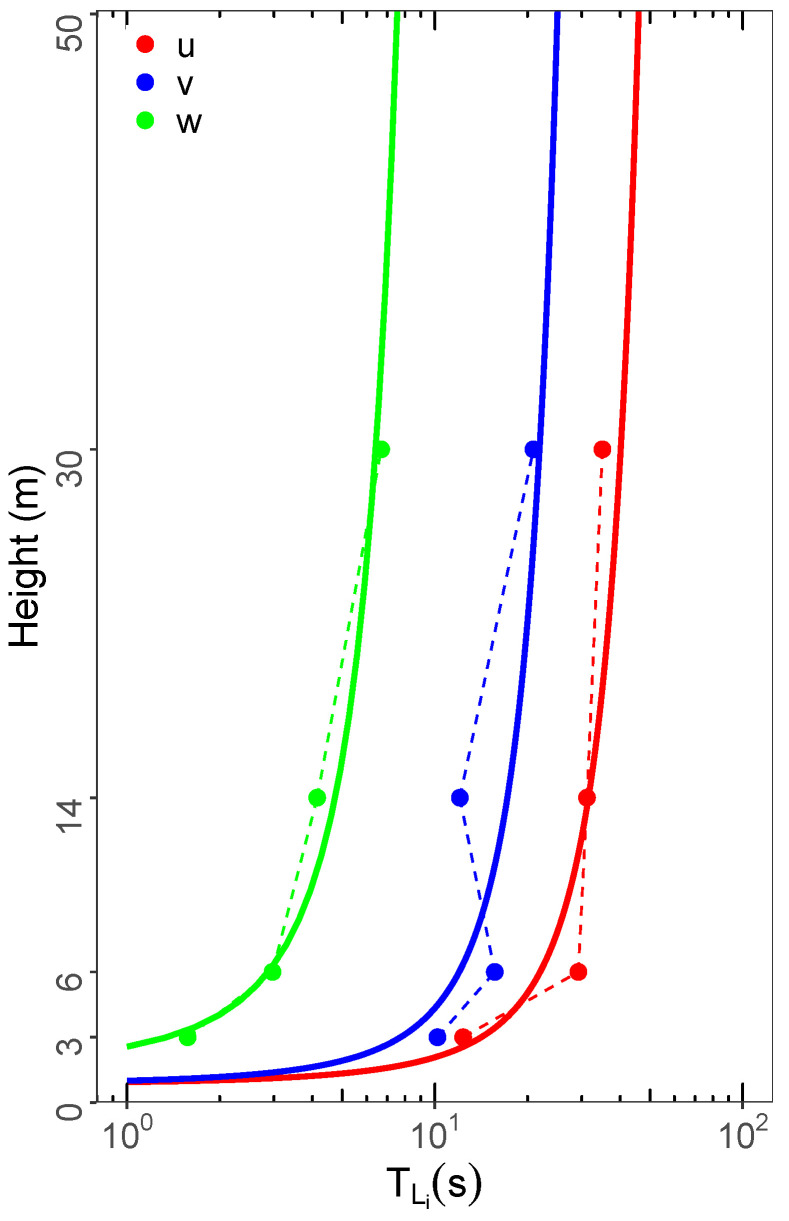
Average vertical profile of the local decorrelation time scales ($T_{L_{i}}$). The dashed and continuous lines represent the values of $T_{L_{i}}$ obtained using Equation (5) and the fit, respectively.

By employing the observed values of $\sigma_{i}$ and ${\left(f_{m}\right)}_{i}$ into Equation (5), the decorrelation time scale’s vertical profiles $T_{L_{i}}$ are obtained (Figure 5). All these scales associated with the turbulent memory effect tend to increase with height. These $T_{L_{i}}$ profiles are fitted by the following expressions: $T_{L_{u}}=11.3\ln1.2z$ (red continuous line), $T_{L_{v}}=5.8\ln1.1z$ (blue continuous line), and $T_{L_{w}}=2.2\ln0.6z$ (green continuous line).

## 5. Simulating Dispersion Experiments

The planetary boundary layer patterns associated with the VNOR episodes are described by persistent northerly winds of moderate-to-strong magnitude and, consequently, for these events, the wind shear forcing mechanism is mainly responsible for a turbulent dispersion generated by dominant mechanical effects. As a consequence, the main features of the turbulent parameterization derived from VNOR episodes is expected to reproduce characteristics of a neutral planetary boundary layer.

In this section, as a test to evaluate the turbulent velocity variances and Lagrangian decorrelation time scales, these parameters are employed in the LAMBDA Lagrangian stochastic particle model to simulate the Prairie Grass neutral dispersion experiments. From the above-mentioned arguments, the classical Prairie Grass tracer dispersion experiments that occurred under *U* > 6 ms${}^{-1}$ and $u_{*}\geq$ 0.4 ms${}^{-1}$ were selected. Following Garrat (1992) [28], these velocity scales characterize a neutral boundary layer. The Prairie Grass tracer release experiments were carried out in O’Neill (Nebraska, USA) in the summer of 1956 [29]. The contaminant (sulfur dioxide, $SO_{2}$) was emitted at the height of 0.5 m on flat ground with a roughness length of 0.6 cm and collected at the height of 1.5 m at the following distances from the source: 50, 100, 200, 400, and 800 m. Table 3 exhibits the main micrometeorological quantities for the 13 selected Prairie Grass runs. In this table, $h=0.2(u_{*}/|f_{c}|)$ is the neutral boundary layer depth, and $f_{c}=10^{-4}$ s${}^{-1}$ is the Coriolis parameter [2,28].

The LAMBDA model is based on the solution of the three-dimensional Langevin equation for the random velocity [30]. The velocity and the displacement of each particle are described by [1]:

(6) $$du_{i}=a_{i}\left(X,u,t\right)+b_{ij}\left(X,u,t\right)dW_{j}\left(t\right)$$

and

(7) dX=(U+u)dt

where X is the displacement vector, U is the mean wind velocity vector, u is the Lagrangian velocity vector, $a_{i}\left(X,u,t\right)$ is a deterministic term, $b_{ij}\left(X,u,t\right)dW_{j}\left(t\right)$ is a stochastic term, and $dW_{j}\left(t\right)$ are the increments of the Wiener process. The LAMBDA solves the stationary Fokker–Planck equation, and it also determines $a_{i}$ and the coefficient $b_{ij}$ through the velocity variances and Lagrangian decorrelation time scales. A detailed description of the LAMBDA model can be found in Ferrero et al. (1995) [31] and Carvalho et al. (2002) [32].

**Figure 6:**
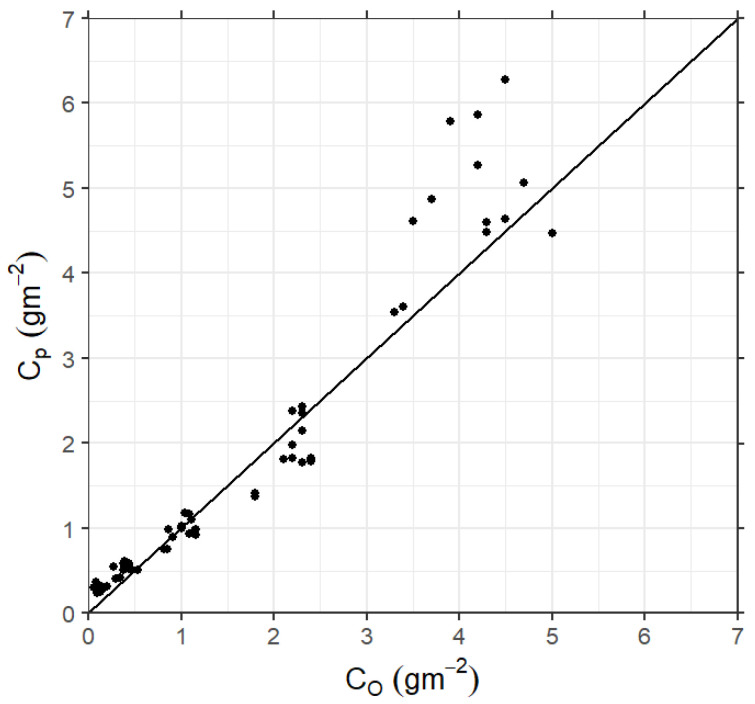
Scatter diagram between observed ($C_{o}$) and predicted ($C_{p}$) ground-level cross-wind integrated concentratios for the Prairie Grass experiment.

The LAMBDA model performance using quasi-experimental $\sigma_{i}$ and $T_{L_{i}}$ obtained from the VNOR cases is presented in Table 4 and Figure 6. Table 4 lists the statistical indices normally employed to compare observed and simulated concentrations. The normalized mean square error (NMSE), fractional bias (FB), and fractional standard deviations (FS) are close to zero and the correlation coefficient (R) close to one. In addition to the statistical indices, Figure 6 exhibits the scatterplot of observed and simulated concentrations.

Therefore, by analyzing the scatter over the central line (Figure 6) and the magnitudes range of the statistical indices (Table 4), it is possible to note that the new parameterization satisfactorily reproduces the observed Prairie Grass concentration data under neutral conditions.

## 6. Conclusions

The mesoscale phenomenon VNOR that has been occurring in southern Brazil has been responsible for establishing, for a long time, a turbulence field driven by a dominant mechanical forcing. This downslope windstorm is characterized by intense and persistent northerly wind gusts associated with a robust advection of warm air, which causes changes in weather patterns. Therefore, a well-established neutral surface boundary layer is frequently observed during the occurrence of a VNOR phenomenon.

High-resolution micrometeorological observations measured during VNOR episodes at the experimental site of the Federal University of Santa Maria (southern Brazil) are used to calculate turbulent dispersion parameters. Peak frequencies extracted from the turbulent energy spectra are identified, and characteristic spatial–temporal scales of the energy-containing eddies are determined. The observational novelty in this study concerns determining the spectral peak frequencies sampled at four levels in a 30 m tower. The dimensional vertical spectral peaks vary as the height increases in the near-surface, while the dimensional horizontal spectral peaks are almost constant in this region. The multi-level measurements allow us to know in more detail the vertical variation of the energy-containing eddy scales responsible for the dominant transport in the surface boundary layer.

The capture of the vertical turbulence’s non-homogeneous character allowed us to determine expressions for the velocity variances, as well as Lagrangian local decorrelation time scales. These dispersion parameters represent the main input quantities to simulate the scalar turbulent transport in a Lagrangian Stochastic Particle Dispersion Model.

To test and evaluate the new neutral turbulent parameterizations, the expressions for $\sigma_{i}$ and $T_{Li}$ were employed in the LAMBDA model to reproduce concentration data of the Prairie Grass experiments. The results showed that the $\sigma_{i}$ and $T_{Li}$ values derived herein may be suitable for applications in dispersion modeling under neutral stability conditions.

## Figures and Tables

**Table 1:** Mean meteorological and micrometeorological variables during the VNOR events.

Height (m)	*U* (ms${}^{-1}$)	$T_{\mathrm{air}}$ (${}^{\circ }$C)	$u_{*}$ (ms${}^{-1}$)	TKE (m${}^{2}$s${}^{-2}$)
3	5.5 ± 1.4	30.3 ± 2.7	0.6 ± 0.2	1.7 ± 0.4
6	6.2 ± 1.5	28.4 ± 2.6	0.7 ± 0.2	1.8 ± 0.4
14	7.1 ± 1.7	28.3 ± 2.6	0.8 ± 0.2	1.9 ± 0.5
30	8.6 ± 1.9	28.6 ± 2.6	0.9 ± 0.2	2.0 ± 0.5

**Table 2:** Non-dimensional ratios between the standard deviation of the wind velocity components and the friction velocity observed at the Santa Maria micrometeorological tower and those found by other studies.

	Height (m)	$\sigma_{u}u_{*}^{-1}$		$\sigma_{v}u_{*}^{-1}$		$\sigma_{w}u_{*}^{-1}$	
		M	O	M	O	M	O
Santa Maria Tower	3	3.2	2.8	3.2	2.3	1.3	1.1
	6	3.6	2.7	2.9	2.4	1.5	1.3
	14	3.0	2.4	2.4	2.0	1.4	1.3
	30	2.5	2.3	2.3	1.8	1.4	1.3
Arbage et al. (2008) [17]	10	2.5	2.7	2.0	1.9	1.4	1.4
Martins et al. (2018) [26]	2	-	2.1	-	1.6	-	1.2
Panofsky and Dutton (1983) [2]	Various	-	2.4	-	1.9	-	1.2
Hanna (1982) [27]	At the surface	-	2.0	-	1.3	-	1.3

**Table 3:** Meteorological parameters during the Prairie Grass experiment.

Run	h (m)	$u_{*}$ (ms${}^{-1}$)	*U* (ms${}^{-1}$)
5	780	0.40	7.0
9	550	0.48	8.4
19	650	0.41	7.2
20	710	0.63	11.3
26	900	0.45	7.8
27	1280	0.44	7.6
30	1560	0.48	8.5
43	600	0.40	6.1
44	1450	0.42	7.2
49	550	0.47	8.0
50	750	0.46	8.0
51	1880	0.47	8.0
61	450	0.53	9.3

**Table 4:** Statistical evaluation of the LAMBDA model.

NMSE	R	FB	FS
0.05	0.96	−0.09	−0.14

## Data Availability

The data used in this study are available by contacting the corresponding author.

## Abbreviations

The following abbreviations are used in this manuscript:

VNOR	Vento Norte (Portuguese for “North Wind”)
TKE	turbulent kinetic energy
NMSE	normalized mean square error
FB	fractional bias
FS	fractional standard deviations
R	correlation coefficient

## References

[1] Rodean H.C. (1996). Stochastic Lagrangian Models of Turbulent Diffusion.

[2] Panofsky H., Dutton J. (1984). Atmospheric Turbulence: Models and Methods for Engineering Applications.

[3] Zannetti P. (1990). Air Pollution Modeling, Computational Mechanics Publications.

[4] Tirabassi T. (2009). Mathematical air pollution models: Eulerian models. Air Pollution and Turbulence: Modeling and Applications.

[5] Anfossi D., Castelli S.T. (2009). An outline of Lagrangian stochastic dispersion models. Air Pollution and Turbulence: Modeling and Applications.

[6] Decker S.G., Robinson D.A. (2011). Unexpected high winds in northern New Jersey: A downslope windstorm in modest topography. Weather. Forecast..

[7] Karmosky C. (2019). Surface Melt on Ross Ice Shelf Interior during a Downsloping Wind Event. Preprints.

[8] Elvidge A.D., Renfrew I.A. (2016). The causes of foehn warming in the lee of mountains. Bull. Am. Meteorol. Soc..

[9] Durran D., North G.R., Pyle J., Zhang F. (2015). Mountain Meteorology|Downslope Winds. Encyclopedia of Atmospheric Sciences.

[10] Whiteman C.D. (2000). Mountain Meteorology: Fundamentals and Applications.

[11] Sartori M. (2003). Gênese e características do vento norte regional em Santa Maria/RS. Anais–X Simpósio Bras. de Geogr. Física Apl..

[12] Anabor V., Acevedo O., Moraes O. (2005). Circulações termicamente induzidas na depressão central do Rio Grande do Sul. Parte I: Intensificação noturna do vento Norte. Cienc. Nat..

[13] Stefanello M., de Lima Nascimento E., da Rosa C.E., Degrazia G., Mortarini L., Cava D. (2020). A Micrometeorological Analysis of the Vento Norte Phenomenon in Southern Brazil. Bound.-Layer Meteorol..

[14] Da Rosa C.E., Stefanello M., de Lima Nascimento E., Rossi F.D., Roberti D.R., Degrazia G.A. (2021). Meteorological observations of the Vento Norte phenomenon in the central region of Rio Grande do Sul. Rev. Bras. Meteorol..

[15] Rubert G., Roberti D., Pereira L., Quadros F., Campos Velho H., Leal de Moraes O. (2018). Evapotranspiration of the Brazilian Pampa Biome: Seasonality and Influential Factors. Water.

[16] Chamis M., Nascimento E. (2012). Condições Atmosféricas Associadas a Episódios de “Vento Norte” na Região Central do RS.

[17] Arbage M.C.A., Degrazia G.A., Welter G.S., Roberti D.R., Acevedo O.C., de Moraes O.L.L., Ferraz S.T., Timm A.U., Moreira V.S. (2008). Turbulent statistical characteristics associated to the north wind phenomenon in southern Brazil with application to turbulent diffusion. Phys. A Stat. Mech. Appl..

[18] Degrazia G., Anfossi D., Carvalho J., Velho H.C., Ferrero E., Mangia C., Rizza U., Castelli S.T. (2000). Turbulence parameterization for PBL dispersion models in all stability conditions. Air Pollution Modeling and Its Application XIII.

[19] Champagne F., Friehe C., LaRue J., Wynagaard J. (1977). Flux measurements, flux estimation techniques, and fine-scale turbulence measurements in the unstable surface layer over land. J. Atmos. Sci..

[20] Sorbjan Z. (1989). Structure of the Atmospheric Boundary Layer.

[21] Boas M.L. (1983). Mathematical Methods in the Physical Sciences.

[22] Gifford F. (1955). A simultaneous Lagrangian-Eulerian Turbulenceexperiment. Mon. Weather Rev..

[23] Wandel C., Kofoed-Hansen O. (1962). On the Eulerian-Lagrangian transform in the statistical theory of turbulence. J. Geophys. Res..

[24] Degrazia G., Anfossi D., Velho H.F.D.C., Ferrero E. (1998). A Lagrangian decorrelation time scale in the convective boundary layer. Bound.-Layer Meteorol..

[25] Kolmogorov A.N. (1941). The local structure of turbulence in incompressible viscous fluid for very large Reynolds numbers. Dokl. Akad. Nauk SSSR. JSTOR.

[26] Martins L.G.N., Degrazia G.A., Acevedo O.C., Puhales F.S., De Oliveira P.E., Teichrieb C.A., Da Silva S.M. (2018). Quasi-Experimental Determination of Turbulent Dispersion Parameters for Different Stability Conditions from a Tall Micrometeorological Tower. J. Appl. Meteorol. Climatol..

[27] Hanna S. (1982). Applications in Air Pollution Modeling. Atmospheric Turbulence and Air Pollution Modeling.

[28] Garratt J. (1992). The Atmospheric Boundary Layer.

[29] Barad M.L. (1958). Project Prairie Grass, a Field Program in Diffusion.

[30] Thomson D. (1987). Criteria for the selection of stochastic models of particle trajectories in turbulent flows. J. Fluid Mech..

[31] Ferrero E., Anfossi D., Brusasca G., Tinarelli G. (1995). Lagrangian particle model LAMBDA: Evaluation against tracer data. Int. J. Environ. Pollut..

[32] Carvalho J.D.C., Degrazia G.A., Anfossi D., De Campos C.R.J., Roberti D.R., Kerr A.S. (2002). Lagrangian stochastic dispersion modelling for the simulation of the release of contaminants from tall and low sources. Meteorol. Z..

